# How Many Species Are There on Earth and in the Ocean?

**DOI:** 10.1371/journal.pbio.1001127

**Published:** 2011-08-23

**Authors:** Camilo Mora, Derek P. Tittensor, Sina Adl, Alastair G. B. Simpson, Boris Worm

**Affiliations:** 1 Department of Biology, Dalhousie University, Halifax, Nova Scotia, Canada; 2 Department of Geography, University of Hawaii, Honolulu, Hawaii, United States of America; 3 United Nations Environment Programme World Conservation Monitoring Centre, Cambridge, United Kingdom; 4 Microsoft Research, Cambridge, United Kingdom; Imperial College London, United Kingdom

## Abstract

The diversity of life is one of the most striking aspects of our planet; hence knowing how many species inhabit Earth is among the most fundamental questions in science. Yet the answer to this question remains enigmatic, as efforts to sample the world's biodiversity to date have been limited and thus have precluded direct quantification of global species richness, and because indirect estimates rely on assumptions that have proven highly controversial. Here we show that the higher taxonomic classification of species (i.e., the assignment of species to phylum, class, order, family, and genus) follows a consistent and predictable pattern from which the total number of species in a taxonomic group can be estimated. This approach was validated against well-known taxa, and when applied to all domains of life, it predicts ∼8.7 million (±1.3 million SE) eukaryotic species globally, of which ∼2.2 million (±0.18 million SE) are marine. In spite of 250 years of taxonomic classification and over 1.2 million species already catalogued in a central database, our results suggest that some 86% of existing species on Earth and 91% of species in the ocean still await description. Renewed interest in further exploration and taxonomy is required if this significant gap in our knowledge of life on Earth is to be closed.

## Author Summary

Knowing the number of species on Earth is one of the most basic yet elusive questions in science. Unfortunately, obtaining an accurate number is constrained by the fact that most species remain to be described and because indirect attempts to answer this question have been highly controversial. Here, we document that the taxonomic classification of species into higher taxonomic groups (from genera to phyla) follows a consistent pattern from which the total number of species in any taxonomic group can be predicted. Assessment of this pattern for all kingdoms of life on Earth predicts ∼8.7 million (±1.3 million SE) species globally, of which ∼2.2 million (±0.18 million SE) are marine. Our results suggest that some 86% of the species on Earth, and 91% in the ocean, still await description. Closing this knowledge gap will require a renewed interest in exploration and taxonomy, and a continuing effort to catalogue existing biodiversity data in publicly available databases.

## Introduction

**Figure 1:**
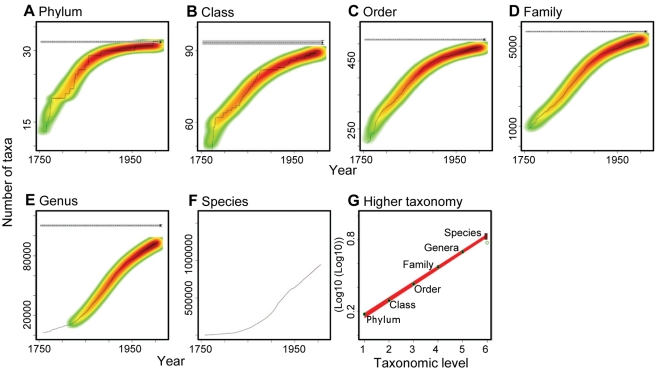
Predicting the global number of species in Animalia from their higher taxonomy. (A–F) The temporal accumulation of taxa (black lines) and the frequency of the multimodel fits to all starting years selected (graded colors). The horizontal dashed lines indicate the consensus asymptotic number of taxa, and the horizontal grey area its consensus standard error. (G) Relationship between the consensus asymptotic number of higher taxa and the numerical hierarchy of each taxonomic rank. Black circles represent the consensus asymptotes, green circles the catalogued number of taxa, and the box at the species level indicates the 95% confidence interval around the predicted number of species (see Materials and Methods).

**Figure 2 pbio-1001127-g002:**
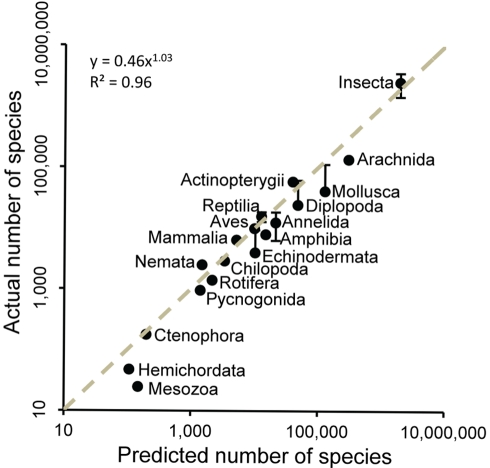
Validating the higher taxon approach. We compared the number of species estimated from the higher taxon approach implemented here to the known number of species in relatively well-studied taxonomic groups as derived from published sources [37]. We also used estimations from multimodel averaging from species accumulation curves for taxa with near-complete inventories. Vertical lines indicate the range of variation in the number of species from different sources. The dotted line indicates the 1∶1 ratio. Note that published species numbers (*y*-axis values) are mostly derived from expert approximations for well-known groups; hence there is a possibility that those estimates are subject to biases arising from synonyms.

Robert May [1] recently noted that if aliens visited our planet, one of their first questions would be, “How many distinct life forms—species—does your planet have?” He also pointed out that we would be “embarrassed” by the uncertainty in our answer. This narrative illustrates the fundamental nature of knowing how many species there are on Earth, and our limited progress with this research topic thus far [1]–[4]. Unfortunately, limited sampling of the world's biodiversity to date has prevented a direct quantification of the number of species on Earth, while indirect estimates remain uncertain due to the use of controversial approaches (see detailed review of available methods, estimates, and limitations in Table 1). Globally, our best approximation to the total number of species is based on the opinion of taxonomic experts, whose estimates range between 3 and 100 million species [1]; although these estimations likely represent the outer bounds of the total number of species, expert-opinion approaches have been questioned due to their limited empirical basis [5] and subjectivity [5]–[6] (Table 1). Other studies have used macroecological patterns and biodiversity ratios in novel ways to improve estimates of the total number of species (Table 1), but several of the underlying assumptions in these approaches have been the topic of sometimes heated controversy ([3]–[17], Table 1); moreover their overall predictions concern only specific groups, such as insects [9],[18]–[19], deep sea invertebrates [13], large organisms [6]–[7],[10], animals [7], fungi [20], or plants [21]. With the exception of a few extensively studied taxa (e.g., birds [22], fishes [23]), we are still remarkably uncertain as to how many species exist, highlighting a significant gap in our basic knowledge of life on Earth. Here we present a quantitative method to estimate the global number of species in all domains of life. We report that the number of higher taxa, which is much more completely known than the total number of species [24], is strongly correlated to taxonomic rank [25] and that such a pattern allows the extrapolation of the global number of species for any kingdom of life (Figures 1 and 2).

**Table 1:** Available methods for estimating the global number of species and their limitations.

Case Study	Limitations
**Macroecological patterns**	
***Body size frequency distributions.*** By extrapolation from the frequency of large to small species, May [7] estimated 10 to 50 million species of animals.	May [7] suggested that there was no reason to expect a simple scaling law from large to small species. Further studies confirmed different modes of evolution among small species [4] and inconsistent body size frequency distributions among taxa [4].
***Latitudinal gradients in species.*** By extrapolation from the better sampled temperate regions to the tropics, Raven [10] estimated 3 to 5 million species of large organisms.	May [2] questioned the assumption that temperate regions were better sampled than tropical ones; the approach also assumed consistent diversity gradients across taxa which is not factual [4].
***Species-area relationships.*** By extrapolation from the number of species in deep-sea samples, Grassle & Maciolek [13] estimated that the world's deep seafloor could contain up to 10 million species.	Lambshead & Bouchet [12] questioned this estimation by showing that high local diversity in the deep sea does not necessarily reflect high global biodiversity given low species turnover.
**Diversity ratios**	
***Ratios between taxa.*** By assuming a global 6∶1 ratio of fungi to vascular plants and that there are ∼270,000 species of vascular plants, Hawksworth [20] estimated 1.6 million fungi species.	Ratio-like approaches have been heavily critiqued because, given known patterns of species turnover, locally estimated ratios between taxa may or may not be consistent at the global scale [3],[12] and because at least one group of organisms should be well known at the global scale, which may not always be true [15]. Bouchet [6] elegantly demonstrated the shortcomings of ratio-based approaches by showing how even for a well-inventoried marine region, the ratio of fishes to total multicellular organisms would yield ∼0.5 million global marine species whereas the ratio of Brachyura to total multicellular organisms in the same sampled region would yield ∼1.5 million species.
***Host-specificity and spatial ratios.*** Given 50,000 known species of tropical trees and assuming a 5∶1 ratio of host beetles to trees, that beetles represent 40% of the canopy arthropods, and that the canopy has twice the species of the ground, Erwin [9] estimated 30 million species of arthropods in the tropics.	
***Known to unknown ratios*** *.* Hodkinson & Casson [18] estimated that 62.5% of the bug (Hemiptera) species in a sampled location were unknown; by assuming that 7.5%–10% of the global diversity of insects is bugs, they estimated between 1.84 and 2.57 million species of insects globally.	
**Taxonomic patterns**	
***Time-species accumulation curves.*** By extrapolation from the discovery record it was estimated that there are ∼19,800 species of marine fishes [23] and ∼11,997 birds [22].	This approach is not widely applicable because it requires species accumulation curves to approach asymptotic levels, which is only true for a small number of well-described taxa [22]–[23].
***Authors-species accumulation curves.*** Modeling the number of authors describing species over time allowed researchers to estimate that the proportion of flowering plants yet to be discovered is 13% to 18% [21].	This is a very recent method and the effect of a number of assumptions remains to be evaluated. One is the extent to which the description of new species is shifting from using taxonomic expertise alone to relying on molecular methods (particularly among small organisms [26]) and the other that not all authors listed on a manuscript are taxonomic experts, particularly in recent times when the number of coauthors per taxa described is increasing [21],[38], which could be due to more collaborative research [38] and the acknowledgment of technicians, field assistants, specimen collectors, and so on as coauthors (Philippe Bouchet, personal communication).
***Analysis of expert estimations.*** Estimates of ∼5 million species of insects [15] and ∼200,000 marine species [14] were arrived at by compiling opinion-based estimates from taxonomic experts. Robustness in the estimations is assumed from the consistency of responses among different experts.	Erwin [5] labeled this approach as “non-scientific” due to a lack of verification. Estimates can vary widely, even those of a single expert [5],[6]. Bouchet [6] argues that expert estimations are often passed on from one expert to another and therefore a robust estimation could be the “same guess copied again and again”.

Higher taxonomy data have been previously used to quantify species richness within specific areas by relating the number of species to the number of genera or families at well-sampled locations, and then using the resulting regression model to estimate the number of species at other locations for which the number of families or genera are better known than species richness (reviewed by Gaston & Williams [24]). This method, however, relies on extrapolation of patterns from relatively small areas to estimate the number of species in other locations (i.e., alpha diversity). Matching the spatial scale of this method to quantify the Earth's total number of species would require knowing the richness of replicated planets; not an option as far as we know, although May's aliens may disagree. Here we analyze higher taxonomic data using a different approach by assessing patterns across all taxonomic levels of major taxonomic groups. The existence of predictable patterns in the higher taxonomic classification of species allows prediction of the total number of species within taxonomic groups and may help to better constrain our estimates of global species richness.

## Results

We compiled the full taxonomic classifications of ∼1.2 million currently valid species from several publicly accessible sources (see Materials and Methods). Among eukaryote “kingdoms,” assessment of the temporal accumulation curves of higher taxa (i.e., the cumulative number of species, genera, orders, classes, and phyla described over time) indicated that higher taxonomic ranks are much more completely described than lower levels, as shown by strongly asymptoting trajectories over time ([24], Figure 1A–1F, Figure S1). However, this is not the case for prokaryotes, where there is little indication of reaching an asymptote at any taxonomic level (Figure S1). For most eukaryotes, in contrast, the rate of discovery of new taxa has slowed along the taxonomic hierarchy, with clear signs of asymptotes for phyla (or “divisions” in botanical nomenclature) on one hand and a steady increase in the number of species on the other (Figure 1A–1F, Figure S1). This prevents direct extrapolation of the number of species from species-accumulation curves [22],[23] and highlights our current uncertainty regarding estimates of total species richness (Figure 1F). However, the increasing completeness of higher taxonomic ranks could facilitate the estimation of the total number of species, if the former predicts the latter. We evaluated this hypothesis for all kingdoms of life on Earth.

First, we accounted for undiscovered higher taxa by fitting, for each taxonomic level from phylum to genus, asymptotic regression models to the temporal accumulation curves of higher taxa (Figure 1A–1E) and using a formal multimodel averaging framework based on Akaike's Information Criterion [23] to predict the asymptotic number of taxa of each taxonomic level (dotted horizontal line in Figure 1A–11E; see Materials and Methods for details). Secondly, the predicted number of taxa at each taxonomic rank down to genus was regressed against the numerical rank, and the fitted models used to predict the number of species (Figure 1G, Materials and Methods). We applied this approach to 18 taxonomic groups for which the total numbers of species are thought to be relatively well known. We found that this approach yields predictions of species numbers that are consistent with inventory totals for these groups (Figure 2). When applied to all eukaryote kingdoms, our approach predicted ∼7.77 million species of animals, ∼298,000 species of plants, ∼611,000 species of fungi, ∼36,400 species of protozoa, and ∼27,500 species of chromists; in total the approach predicted that ∼8.74 million species of eukaryotes exist on Earth (Table 2). Restricting this approach to marine taxa resulted in a prediction of 2.21 million eukaryote species in the world's oceans (Table 2). We also applied the approach to prokaryotes; unfortunately, the steady pace of description of taxa at all taxonomic ranks precluded the calculation of asymptotes for higher taxa (Figure S1). Thus, we used raw numbers of higher taxa (rather than asymptotic estimates) for prokaryotes, and as such our estimates represent only lower bounds on the diversity in this group. Our approach predicted a lower bound of ∼10,100 species of prokaryotes, of which ∼1,320 are marine. It is important to note that for prokaryotes, the species concept tolerates a much higher degree of genetic dissimilarity than in most eukaryotes [26],[27]; additionally, due to horizontal gene transfers among phylogenetic clades, species take longer to isolate in prokaryotes than in eukaryotes, and thus the former species are much older than the latter [26],[27]; as a result the number of described species of prokaryotes is small (only ∼10,000 species are currently accepted).

**Table 2:** Currently catalogued and predicted total number of species on Earth and in the ocean.

Species	Earth			Ocean		
	Catalogued	Predicted	±SE	Catalogued	Predicted	±SE
**Eukaryotes**						
Animalia	953,434	7,770,000	958,000	171,082	2,150,000	145,000
Chromista	13,033	27,500	30,500	4,859	7,400	9,640
Fungi	43,271	611,000	297,000	1,097	5,320	11,100
Plantae	215,644	298,000	8,200	8,600	16,600	9,130
Protozoa	8,118	36,400	6,690	8,118	36,400	6,690
*Total*	1,233,500	8,740,000	1,300,000	193,756	2,210,000	182,000
**Prokaryotes**						
Archaea	502	455	160	1	1	0
Bacteria	10,358	9,680	3,470	652	1,320	436
*Total*	10,860	10,100	3,630	653	1,320	436
**Grand Total**	**1,244,360**	**8,750,000**	**1,300,000**	**194,409**	**2,210,000**	**182,000**

Predictions for prokaryotes represent a lower bound because they do not consider undescribed higher taxa. For protozoa, the ocean database was substantially more complete than the database for the entire Earth so we only used the former to estimate the total number of species in this taxon. All predictions were rounded to three significant digits.

### Assessment of Possible Limitations

We recognize a number of factors that can influence the interpretation and robustness of the estimates derived from the method described here. These are analyzed below.

#### Species definitions

An important caveat to the interpretation of our results concerns the definition of species. Different taxonomic communities (e.g., zoologists, botanists, and bacteriologists) use different levels of differentiation to define a species. This implies that the numbers of species for taxa classified according to different conventions are not directly comparable. For example, that prokaryotes add only 0.1% to the total number of known species is not so much a statement about the diversity of prokaryotes as it is a statement about what a species means in this group. Thus, although estimates of the number of species are internally consistent for kingdoms classified under the same conventions, our aggregated predictions for eukaryotes and prokaryotes should be interpreted with that caution in mind.

#### Changes in higher taxonomy

**Figure 3:**
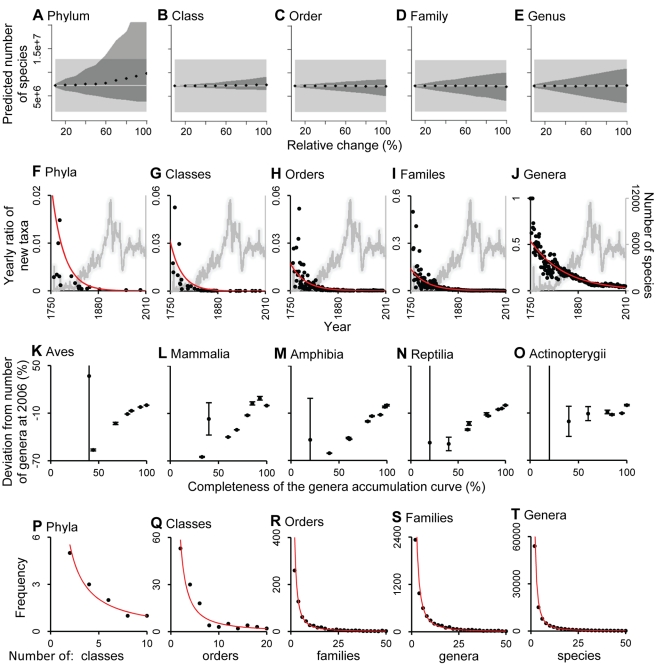
Assessment of factors affecting the higher taxon approach. (A–E) To test the effects of changes in higher taxonomy, we performed a sensitivity analysis in which the number of species was calculated after altering the number of higher taxa. We used Animalia as a test case. For each taxonomic level, we added or removed a random proportion of taxa from 10% to 100% of the current number of taxa and recalculated the number of species using our method. The test was repeated 1,000 times and the average and 95% confidence limits of the simulations are shown as points and dark areas, respectively. Light gray lines and boxes indicate the currently estimated number of species and its 95% prediction interval, respectively. Our current estimation of the number of species appear robust to changes in higher taxonomy as in most cases changes in higher taxonomy led to estimations that remained within the current estimated number of species. The results for changes in all possible combinations of taxonomic levels are shown in Figure S2. (F–J) The yearly ratio of new higher taxa in Animalia (black points and red line) and the yearly number of new species (grey line); this reflects the fraction of newly described species that also represent new higher taxa. The contrasting patterns in the description of new species and new higher taxa suggest that taxonomic effort is probably not driving observed flattening of accumulation curves in higher taxonomic levels as there is at least sufficient effort to maintain a constant description of new species. (K–O) Sensitivity analysis on the completeness of taxonomic inventories. To assess the extent to which incomplete inventories affect the predicted consensus asymptotic values obtained from the temporal accumulation of taxa, we performed a sensitivity analysis in which the consensus asymptotic number of taxa was calculated from curves at different levels of completeness. We used the accumulation curves at the genus level for major groups of vertebrates, given the relative completeness of these data (i.e., reaching an asymptote). Vertical lines indicate the consensus standard error. (P–T) Frequency distribution of the number of subordinate taxa at different taxonomic levels. For display purposes we present only the data for Animalia; lines and test statistics are from a regression model fitted with a power function.

Increases or decreases in the number of higher taxa will affect the raw data used in our method and thus its estimates of the total number of species. The number of higher taxa can change for several reasons including new discoveries, the lumping or splitting of taxa due to improved phylogenies and switching from phenetic to phylogenetic classifications, and the detection of synonyms. A survey of 2,938 taxonomists with expertise across all major domains of life (response rate 19%, see Materials and Methods) revealed that synonyms are a major problem at the species level, but much less so at higher taxonomic levels. The percentage of taxa names currently believed to be synonyms ranged from 17.9 (±28.7 SD) for species, to 7.38 (±15.8 SD) for genera, to 5.5 (±34.0 SD) for families, to 3.72 (±45.2 SD) for orders, to 1.15 (±8.37 SD) for classes, to 0.99 (±7.74 SD) for phyla. These results suggest that by not using the species-level data, our higher-taxon approach is less sensitive to the problem of synonyms. Nevertheless, to assess the extent to which any changes in higher taxonomy will influence our current estimates, we carried out a sensitivity analysis in which the number of species was calculated in response to variations in the number of higher taxa (Figure 3A–3E, Figure S2). This analysis indicates that our current estimates are remarkably robust to changes in higher taxonomy.

#### Changes in taxonomic effort

Taxonomic effort can be a strong determinant of species discovery rates [21]. Hence the estimated asymptotes from the temporal accumulation curves of higher taxa (dotted horizontal line in Figure 1A–1E) might be driven by a decline in taxonomic effort. We presume, however, that this is not a major factor: while the discovery rate of higher taxa is declining (black dots and red lines in Figure 3F–3J), the rate of description of new species remains relatively constant (grey lines in Figure 3F–3J). This suggests that the asymptotic trends among higher taxonomic levels do not result from a lack of taxonomic effort as there has been at least sufficient effort to describe new species at a constant rate. Secondly, although a majority (79.4%) of experts that we polled in our taxonomic survey felt that the number of taxonomic experts is decreasing, it was pointed out that other factors are counteracting this trend. These included, among others, more amateur taxonomists and phylogeneticists, new sampling methods and molecular identification tools, increased international collaboration, better access to information, and access to new areas of exploration. Taken together these factors have resulted in a constant rate of description of new species, as evident in our Figure 1, Figure 3F–3J, and Figure S1 and suggest that the observed flattening of the discovery curves of higher taxa is unlikely to be driven by a lack of taxonomic effort.

#### Completeness of taxonomic inventories

To account for yet-to-be-discovered higher taxa, our approach fitted asymptotic regression models to the temporal accumulation curve of higher taxa. A critical question is how the completeness of such curves will affect the asymptotic prediction. To address this, we performed a sensitivity analysis in which the asymptotic number of taxa was calculated for accumulation curves with different levels of completeness. The results of this test indicated that the asymptotic regression models used here would underestimate the number of predicted taxa when very incomplete inventories are used (Figure 3K–3O). This underestimation in the number of higher taxa would lower our prediction of the number of species through our higher taxon approach, which suggests that our species estimates are conservative, particularly for poorly sampled taxa. We reason that underestimation due to this effect is severe for prokaryotes due to the ongoing discovery of higher taxa (Figure S1) but is likely to be modest in most eukaryote groups because the rate of discovery of higher taxa is rapidly declining (Figure 1A–[Fig pbio-1001127-g002] 3E, Figure S1, Figure 3F–3J).

Since higher taxonomic levels are described more completely (Figure 1A–1E), the resulting error from incomplete inventories should decrease while rising in the taxonomic hierarchy. Recalculating the number of species while omitting all data from genera yielded new estimates that were mostly within the intervals of our original estimates (Figure S3). However, Chromista (on Earth and in the ocean) and Fungi (in the ocean) were exceptions, having inflated predictions without the genera data (Figure S3). This inflation in the predicted number of species without genera data highlights the high incompleteness of at least the genera data in those three cases. In fact, Adl et al.'s [28] survey of expert opinions reported that the number of described species of chromists could be in the order of 140,000, which is nearly 10 times the number of species currently catalogued in the databases used here (Table 1). These results suggest that our estimates for Chromista and Fungi (in the ocean) need to be considered with caution due to the incomplete nature of their data.

#### Subjectivity in the Linnaean system of classification

Different ideas about the correct classification of species into a taxonomic hierarchy may distort the shape of the relationships we describe here. However, an assessment of the taxonomic hierarchy shows a consistent pattern; we found that at any taxonomic rank, the diversity of subordinate taxa is concentrated within a few groups with a long tail of low-diversity groups (Figure 3P–3T). Although we cannot refute the possibility of arbitrary decisions in the classification of some taxa, the consistent patterns in Figure 3P–3T imply that these decisions do not obscure the robust underlying relationship between taxonomic levels. The mechanism for the exponential relationships between nested taxonomic levels is uncertain, but in the case of taxa classified phylogenetically, it may reflect patterns of diversification likely characterized by radiations within a few clades and little cladogenesis in most others [29]. We would like to caution that the database we used here for protistan eukaryotes (mostly in Protozoa and Chromista in this work) combines elements of various classification schemes from different ages—in fact the very division of these organisms into “Protozoa” and “Chromista” kingdoms is non-phylogenetic and not widely followed among protistologists [28]. It would be valuable to revisit the species estimates for protistan eukaryotes once their global catalogue can be organized into a valid and stable higher taxonomy (and their catalogue of described species is more complete—see above).

## Discussion

Knowing the total number of species has been a question of great interest motivated in part by our collective curiosity about the diversity of life on Earth and in part by the need to provide a reference point for current and future losses of biodiversity. Unfortunately, incomplete sampling of the world's biodiversity combined with a lack of robust extrapolation approaches has yielded highly uncertain and controversial estimates of how many species there are on Earth. In this paper, we describe a new approach whose validation against existing inventories and explicit statistical nature adds greater robustness to the estimation of the number of species of given taxa. In general, the approach was reasonably robust to various caveats, and we hope that future improvements in data quality will further diminish problems with synonyms and incompleteness of data, and lead to even better (and likely higher) estimates of global species richness.

Our current estimate of ∼8.7 million species narrows the range of 3 to 100 million species suggested by taxonomic experts [1] and it suggests that after 250 years of taxonomic classification only a small fraction of species on Earth (∼14%) and in the ocean (∼9%) have been indexed in a central database (Table 2). Closing this knowledge gap may still take a lot longer. Considering current rates of description of eukaryote species in the last 20 years (i.e., 6,200 species per year; ±811 SD; Figure 3F–3J), the average number of new species described per taxonomist's career (i.e., 24.8 species, [30]) and the estimated average cost to describe animal species (i.e., US$48,500 per species [30]) and assuming that these values remain constant and are general among taxonomic groups, describing Earth's remaining species may take as long as 1,200 years and would require 303,000 taxonomists at an approximated cost of US$364 billion. With extinction rates now exceeding natural background rates by a factor of 100 to 1,000 [31], our results also suggest that this slow advance in the description of species will lead to species becoming extinct before we know they even existed. High rates of biodiversity loss provide an urgent incentive to increase our knowledge of Earth's remaining species.

Previous studies have indicated that current catalogues of species are biased towards conspicuous species with large geographical ranges, body sizes, and abundances [4],[32]. This suggests that the bulk of species that remain to be discovered are likely to be small-ranged and perhaps concentrated in hotspots and less explored areas such as the deep sea and soil; although their small body-size and cryptic nature suggest that many could be found literally in our own “backyards” (after Hawksworth and Rossman [33]). Though remarkable efforts and progress have been made, a further closing of this knowledge gap will require a renewed interest in exploration and taxonomy by both researchers and funding agencies, and a continuing effort to catalogue existing biodiversity data in publicly available databases.

## Materials and Methods

### Databases

Calculations of the number of species on Earth were based on the classification of currently valid species from the Catalogue of Life (www.sp2000.org, [34]) and the estimations for species in the ocean were based on The World's Register of Marine Species (www.marinespecies.org, [35]). The latter database is largely contained within the former. These databases were screened for inconsistencies in the higher taxonomy including homonyms and the classification of taxa into multiple clades (e.g., ensuring that all diatom taxa were assigned to “Chromista” and not to “plants”). The Earth's prokaryotes were analyzed independently using the most recent classification available in the List of Prokaryotic Names with Standing in Nomenclature database (http://www.bacterio.cict.fr). Additional information on the year of description of taxa was obtained from the Global Names Index database (http://www.globalnames.org). We only used data to 2006 to prevent artificial flattening of accumulation curves due to recent discoveries and descriptions not yet being entered into databases.

### Statistical Analysis

To account for higher taxa yet to be discovered, we used the following approach. First, for each taxonomic rank from phylum to genus, we fitted six asymptotic parametric regression models (i.e., negative exponential, asymptotic, Michaelis-Menten, rational, Chapman-Richards, and modified Weibull [23]) to the temporal accumulation curve of higher taxa (Figure 1A–1E) and used multimodel averaging based on the small-sample size corrected version of Akaike's Information Criteria (AIC_c_) to predict the asymptotic number of taxa (dotted horizontal line in Figure 1A–1E) [23]. Ideally data should be modeled using only the decelerating part of the accumulation curve [22]–[23], however, frequently there was no obvious breakpoint at which accumulation curves switched from an increasing to a decelerating rate of discovery (Figure 1A–1E). Therefore, we fitted models to data starting at all possible years from 1758 onwards (data before 1758 were added as an intercept to prevent a spike due to Linnaeus) and selected the model predictions if at least 10 years of data were available and if five of the six asymptotic models converged to the subset data. Then, the estimated multimodel asymptotes and standard errors for each selected year were used to estimate a consensus asymptote and its standard error. In this approach, the multimodel asymptotes for all cut-off years selected and their standard errors are weighted proportionally to their standard error, thus ensuring that the uncertainty both within and among predictions were incorporated [36].

To estimate the number of species in a taxonomic group from its higher taxonomy, we used Least Squares Regression models to relate the consensus asymptotic number of higher taxa against their numerical rank, and then used the resulting regression model to extrapolate to the species level (Figure 1G). Since data are not strictly independent across hierarchically organized taxa, we also used models based on Generalized Least Squares assuming autocorrelated regression errors. Both types of models were run with and without the inverse of the consensus estimate variances as weights to account for differences in certainty in the asymptotic number of higher taxa. We evaluated the fit of exponential, power, and hyperexponential functions to the data and obtained a prediction of the number of species by multimodel averaging based on AIC_c_ of the best type of function. The hyperexponential function was chosen for kingdoms whereas the exponential function for the smaller groups was used in the validation analysis (see comparison of fits in Figure S4).

### Survey of Taxonomists

We contacted 4,771 taxonomy experts with electronic mail addresses as listed in the World Taxonomist Database (www.eti.uva.nl/tools/wtd.php); 1,833 were faulty e-mails, hence about 2,938 experts received our request, of which 548 responded to our survey (response rate of 18.7%). Respondents were asked to identify their taxon of expertise, and to comment on what percentage of currently valid names could be synonyms at taxonomic levels from species to kingdom. We also polled taxonomists about whether the taxonomic effort (measured as numbers of professional taxonomists) in their area of expertise in recent times was increasing, decreasing, or stable.

## Supporting Information

Figure S1 Completeness of the higher taxonomy of kingdoms of life on Earth.
(DOC)

Figure S2 Sensitivity analysis due to changes in higher taxonomy.
(DOC)

Figure S3 Assessing the effects of data incompleteness.
(DOC)

Figure S4 Comparison of the fits of the hyperexponential, exponential, and power functions to the relationship between the number of higher taxa and their numerical rank.
(DOC)
