# Supplementary material for: How Many Species Are There on Earth and in the Ocean?
Source: PLoS Biol. 2011 Aug 23;9(8):e1001127. doi: 10.1371/journal.pbio.1001127 (PMC3160336; doi:10.1371/journal.pbio.1001127)

**Figure S1. Completeness of the higher taxonomy of kingdoms of life on Earth.** Columns 1 to 6 indicate the temporal accumulation of the number of taxa at each taxonomic rank (blue lines). The horizontal red lines indicate the consensus mean on the number of taxa (see Methods). The plots on the far right show the relationship between the number of taxa and the numerical rank (y-axis is in double log10). Vertical red areas indicate the 95% prediction interval on the number of species. Blue symbols indicate the currently cataloged number of taxa and red symbols the consensus mean. Where only blue symbols are shown the catalogued and consensus means overlapped. Note that predictions of the number of species are based on weighted averages of multiple models (see Materials and Methods).


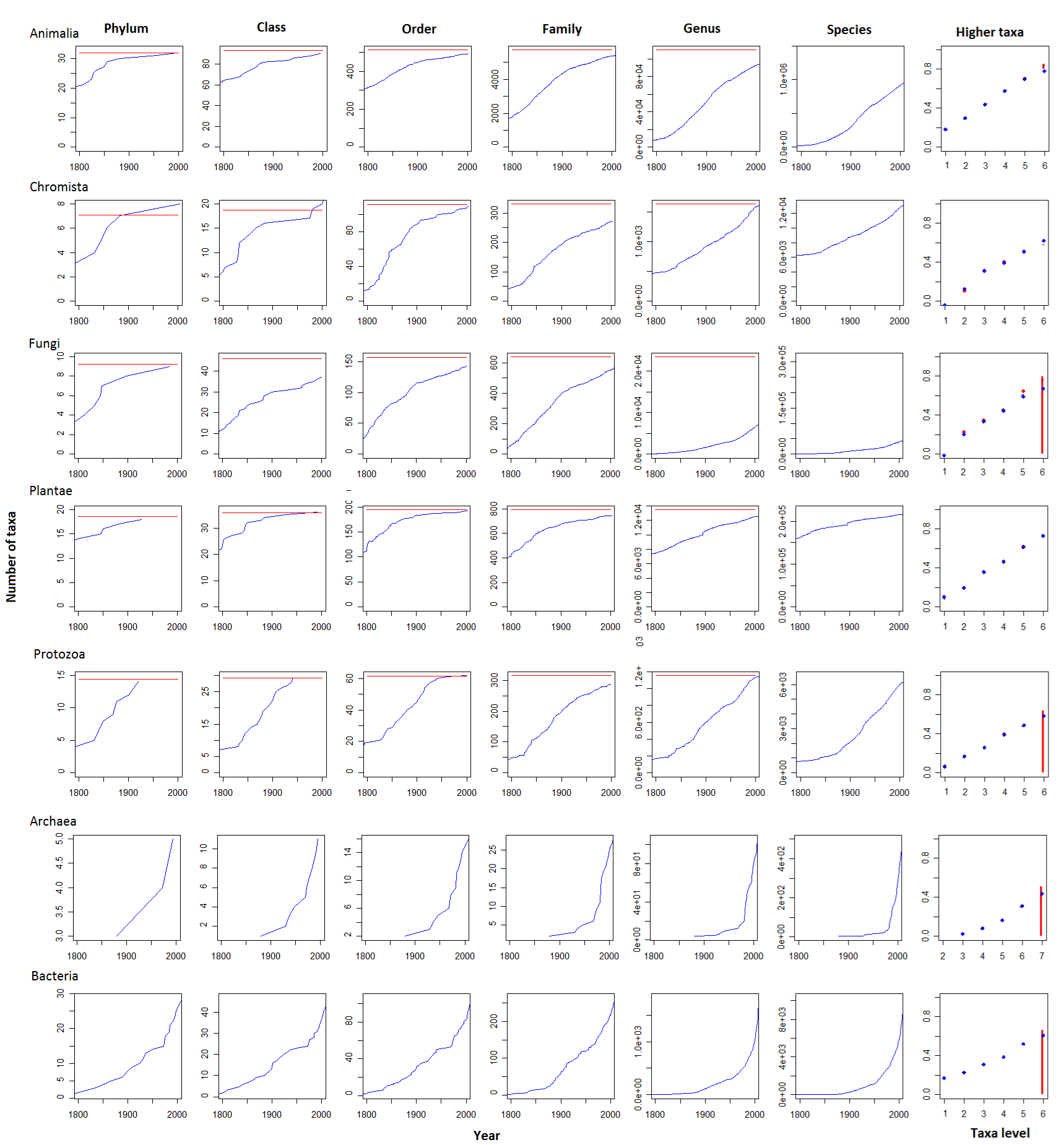


Cont. Figure S1. Completeness of the higher taxonomy of kingdoms of life in the ocean.


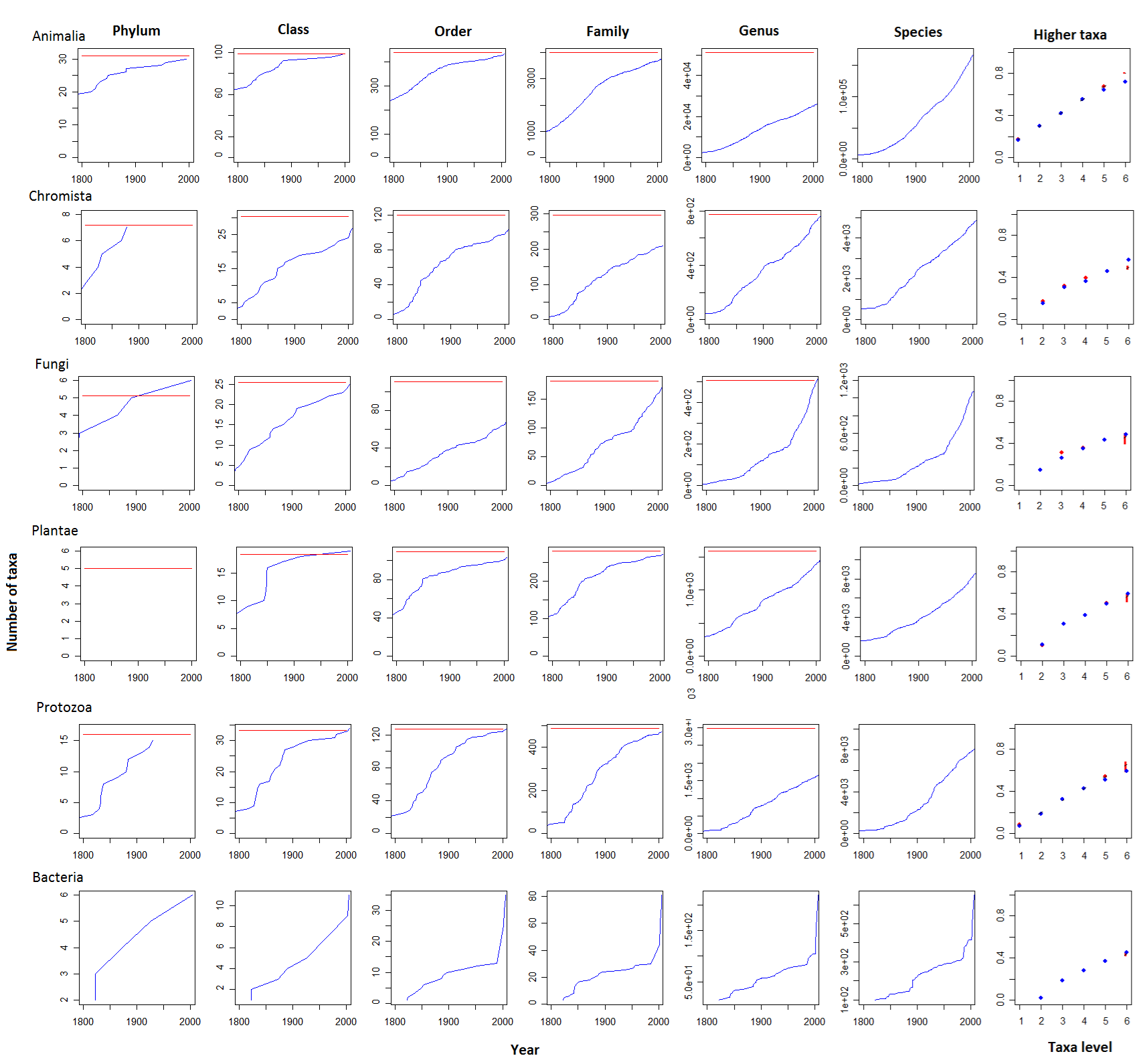

Supplement: Figure S1 — Completeness of the higher taxonomy of kingdoms of life on Earth. (DOC) [file pbio.1001127.s001.doc]
