# Supplementary material for: How Many Species Are There on Earth and in the Ocean?
Source: PLoS Biol. 2011 Aug 23;9(8):e1001127. doi: 10.1371/journal.pbio.1001127 (PMC3160336; doi:10.1371/journal.pbio.1001127)
Supplement: Figure S2 — Sensitivity analysis due to changes in higher taxonomy. (DOC) [file pbio.1001127.s002.doc]

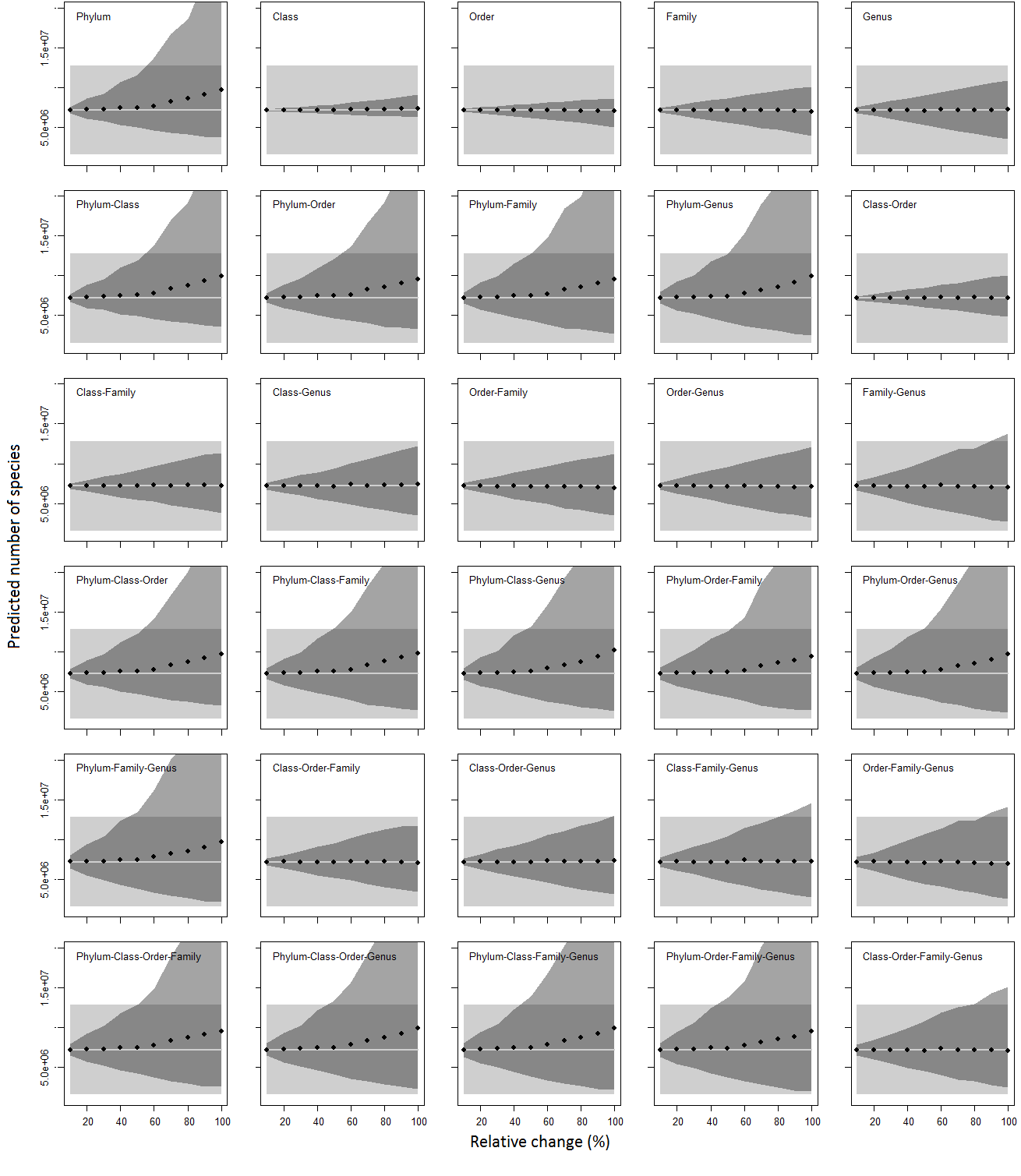
**Figure S2. Sensitivity analysis due to changes in higher taxonomy.** One potential caveat in our approach is the degree to which estimations of the number of species may change if higher taxonomy changes. To test this effect we performed a sensitivity analysis in which the number of species was calculated after altering the number of higher taxa. We used Animalia as a test case. For each possible combination of taxonomic levels, we added or removed a random proportion of taxa from 10 to 100% of the current number of taxa and recalculated the number of species using the method described in this paper. The approach was repeated 1000 times and the average and 95% confidence limits of the simulations are shown as points and dark areas, respectively. Light gray lines and boxes indicate the currently estimated number of species and its 95% prediction interval, respectively. Our current estimation of the number of species appear remarkably robust to changes in higher taxonomy. As noted, results were only sensitive to changes in the number of phyla, and only when this number changed by over 50% relative to the number of currently described phyla. For all other taxonomic levels, expected changes led to predictions that remain within the confidence intervals of the currently estimated number of species.
