# Supplementary material for: How Many Species Are There on Earth and in the Ocean?
Source: PLoS Biol. 2011 Aug 23;9(8):e1001127. doi: 10.1371/journal.pbio.1001127 (PMC3160336; doi:10.1371/journal.pbio.1001127)
Supplement: Figure S3 — Assessing the effects of data incompleteness. (DOC) [file pbio.1001127.s003.doc]

F**igure S3. Assessing the effects of data incompleteness.** Since higher taxonomic levels are described more completely (Figure 1A-E), the resulting error from incomplete inventories should decrease while rising in the taxonomic hierarchy. Therefore, rerunning our higher taxon approach without lower taxonomic ranks could allow to spot kingdoms for which incomplete inventories could be an issue. Thus, we recalculated the number of species using our higher taxon approach without the number of genera. We did not remove higher levels because this led to considerable less data whose range was farther away from the level to predict and this is known to the yield erratic predictions and large errors. The new estimates without genera data were within the intervals of our original estimates for most kingdoms. However, chromista (on Earth and in the ocean) and fungi (in the ocean) were exceptions, having inflated predictions without the genera data (see plots below). The fact that the genera data has a strong effect in those three cases likely reflects the fact that the genus data on those cases are considerably incomplete and therefore drags down the predicted number of species when the genus data are considered. We are aware that the data for chromista currently catalogued in the database we used is very incomplete (e.g. Adl et al *[1]*, surveying expert opinions, reported that the number of described species of chromista could be in the order of 140,000 species, while the number currently catalogued is not more than 14.000, see Table 1). These results suggest that incomplete inventories do have an important effect for chromista and fungi in the ocean and that our predictions will improve as a more complete database becomes available for those groups. Error bars indicate standard errors. The red line indicates the 1:1 ratio.


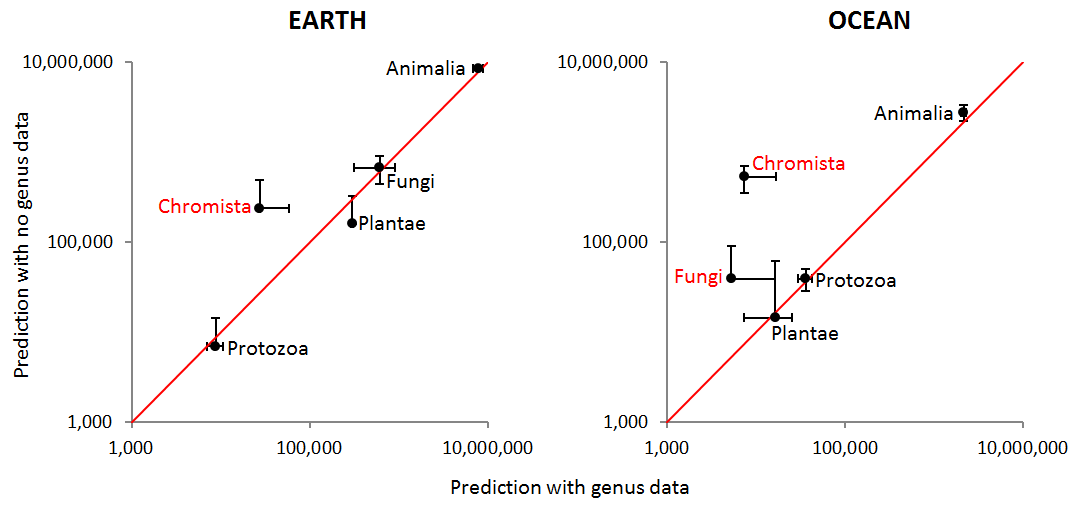


***References***

1. Adl SM, Leander BS, Simpson AGB, Archibald JM, Anderson OR, et al. (2007) Diversity, nomenclature, and taxonomy of protists. *Syst Biol* 56: 684-689.
