# Supplementary material for: How Many Species Are There on Earth and in the Ocean?
Source: PLoS Biol. 2011 Aug 23;9(8):e1001127. doi: 10.1371/journal.pbio.1001127 (PMC3160336; doi:10.1371/journal.pbio.1001127)
Supplement: Figure S4 — Comparison of the fits of the hyperexponential, exponential, and power functions to the relationship between the number of higher taxa and their numerical rank. (DOC) [file pbio.1001127.s004.doc]

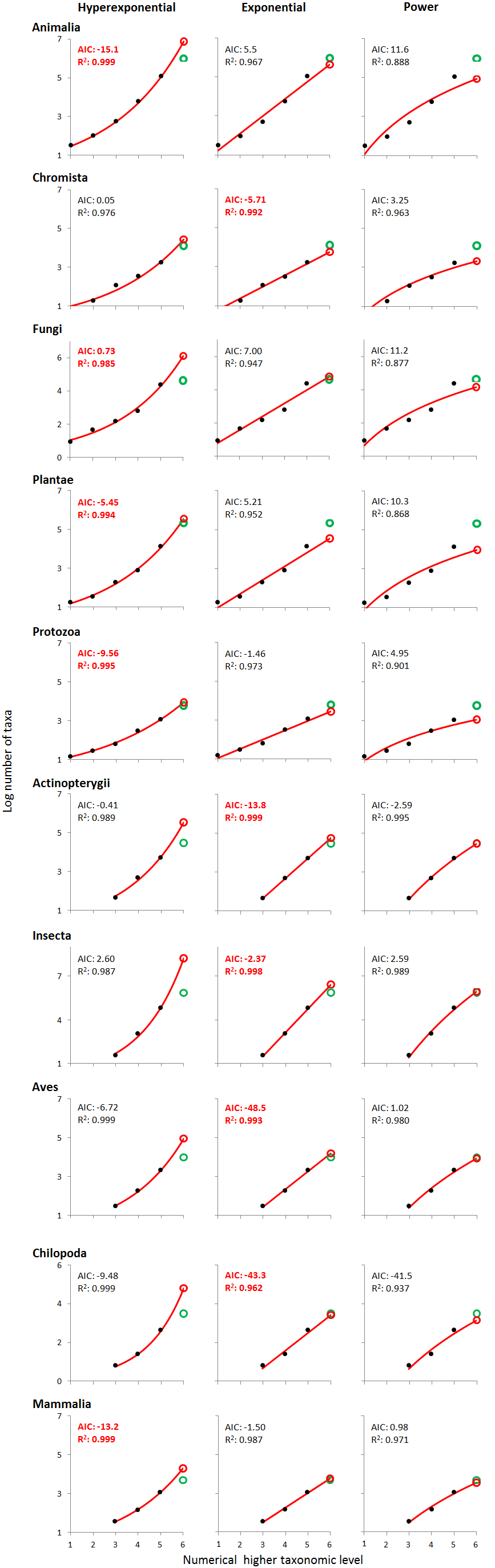
**Figure S4. Comparison of the fits of the hyperexponential, exponential and power functions to the relationship between the number of higher taxa and their numerical rank.** For all kingdoms but Chromista, the hyperexponential function was chosen as the best function whereas the exponential function was commonly the best one among the smaller groups used in the validation approach. Hyperexponential functions and their analogous hyperbolic functions have been recently used to model temporal trends in the size of the world’s human population *[1]* and evolutionary rates of biological diversification *[2]*. These functions are used to describe self-accelerating processes and patterns during a long period in which there is a transition from zero growth to the exponential one; these same patterns can also be seen as a long term sequence of exponential steps *[3]*. We presume then that the variable importance of hyperexponential and exponential functions between kingdoms and smaller groups likely obeys to the fact that the latter uses a very restricted set of data (a short “step” of the pattern) and therefore it is better described with the exponential function. We are aware that the data for Chromista is significantly incomplete (see section of Caveats), and therefore, the selection of a different model to the hyperexponential in that case may well be due to incomplete data. The higher taxonomy of this group is also not fully agreed upon *[4]*. So to prevent errors in the incorrect selection of models due to data inaccuracy, we assume that the relationship between the number of higher taxa and their numerical rank follows a hyperexponential pattern and therefore as a rule of thumb we used the hyperexponential function for all kingdoms whereas the exponential function was used for the validation groups due to the small subset of their data. Using multimodel averaging of all functions combined yielded almost identical results for all kingdoms but Chromista. For Chromista, the selection of the exponential function weighted the final prediction in favour of the estimates of that function yielding a predicted number of species of 5,770 (+/- 26 SE), which is a clear underestimation of the number of species in this group and which reasonably can be argued is due to the wrong model selected. Red circles indicate the predicted number of species whereas the green circle the currently catalogued number of species.

***References***

1. Varfolomeev SD, Gurevich KD (2001) The hyperexponential growth of the human population on a macrohistorical scale. J. Theor. Biol. 212: 367-372.

2. Markov AV, Korotayev AV (2007) Phanerozoic marine biodiversity follows a hyperbolic trend. Palaeoworld 16: 311–318.

3. Hanson R (1998) Long-Term Growth as a Sequence of Exponential Modes, <http://hanson.gmu.edu/longgrow.pdf>.

4. Adl SM, Leander BS, Simpson AGB, Archibald JM, Anderson OR, et al. (2007) Diversity, nomenclature, and taxonomy of protists. *Syst Biol* 56: 684-689.
